# Supplementary material for: Healthcare System Distrust and Non-Prescription Antibiotic Use: A Cross-Sectional Survey of Adult Antibiotic Users
Source: Antibiotics (Basel). 2023 Jan 1;12(1):79. doi: 10.3390/antibiotics12010079 (PMC9854942; doi:10.3390/antibiotics12010079)
Supplement: Supplementary file 1 [file antibiotics-12-00079-s001.zip › antibiotics-2101759-supplementary.pdf]

Supplementary Material

Study Survey

The English version of the full survey used for this study is listed below: (survey was available in both English and Spanish)

Section 1: Screening Questions

1. What is your country of primary residence?
    - United States
    - Mexico
2. In which state do you currently reside?
3. What is the Zip Code of your primary residence?
4. In which state do you currently reside?

Section 2: Past use of Antibiotics

5. Have you ever taken the following antibiotics? (check all that apply)
    - Oral antibiotics
    - Injectable antibiotics
    - Topical antibiotics (gel applied to skin, or drops to eyes or ears)
    - I have never taken any type of antibiotics
6. Where did you obtain the topical antibiotic and for what purpose were you taking it?
7. What type of injectable antibiotic have you used?
    - intramuscular (into a muscle)
    - intravenous (into a vein)
8. Where did you obtain this injectable antibiotic?
    - Hospital or clinic setting
    - Non-hospital setting. Please best describe the setting where you obtained this injectable antibiotic.
9. Who administered the injectable antibiotic?
    - Self-administered
    - A healthcare professional
    - A family member or friend
10. Where did you obtain this injectable antibiotic?
    - Medical clinic
    - My own supply leftover from previous sickness
    - Neighborhood grocery store
    - Family member or friend
    - Internet pharmacy, or online
    - Community pharmacy
    - Flea market
    - Swap meet
    - Veterinarian's office or supply store
    - Bodega
    - Health Store

- Some other source not listed

11. When was the last time you took oral antibiotics outside of a hospital setting?
    - Within the past month
    - Within the past 3 months
    - In the last 6 months
    - In the last year
    - More than a year ago
    - Never
12. On that (most recent) occasion, where did you obtain the antibiotics?
    - From a doctor or nurse at a medical office
    - My own supply leftover from previous sickness
    - Neighborhood grocery store
    - Family member or friend
    - Internet pharmacy, or online
    - Community pharmacy
    - Flea market
    - Swap meet
    - Veterinarian's office or supply store
    - Bodega
    - Health Store
    - Some other source not listed

Section 3: Self-reported Behaviors related to Antibiotic Use

Please answer the following true or false questions. If unsure of the answer, please select the option which you feel is most true.

13. I have oral or injectable antibiotics at home right now, including oral antibiotics I am currently using or have recently used. (True/False)
14. I have asked my healthcare provider to prescribe me, my child, or parent oral antibiotics within the past 3 years. (True/False)
15. I have bought non-prescription oral or injectable antibiotics within the past 3 years. (True/False)
16. I have asked my friends or family for leftover oral or injectable antibiotics within the past 3 years. (True/False)
17. I am more likely to seek out access to self-prescribed oral or injectable antibiotics for my child or parent than for myself I feel as though I have good access to healthcare (True/False)
18. Would you travel to Mexico for antibiotics if they were too difficult to obtain in the United States? (Yes/No)
19. How long would you be willing to travel to obtain antibiotics?
    - 30 min or less
    - More than 30 min
    - More than an hour
20. I would travel to obtain antibiotics because (choose all that apply):
    - They are difficult to obtain in the US
    - Because of the price in the US
21. This is a question to make sure you are a human. Please select "C".

- A
- B
- C
- D

Section 4: Knowledge about Antibiotic Use

22. I would try to get antibiotics if I had the following symptoms: (Check all that apply)
    - Burning/Discomfort while urinating
    - Fatigue
    - Cloudy, dark, or bloody urination
    - Sore Throat
    - Stomach Ache
    - Abdominal Pain
    - Cough
    - Fever
    - Ear Pain
    - Rash
    - Diarrhea
    - Genital Itching/Irritation
    - Genital Burning
    - Difficulty Breathing
    - Tooth Pain
    - Nausea
    - Muscle Aches
    - Runny nose
    - Vomiting
    - Loss of smell or taste
    - None of the above
    - Other
23. Which of the following can be treated with antibiotics? Answer to the best of your ability. Check all that apply
    - HIV/AIDS
    - Gonorrhea
    - Bladder infection OR urinary tract infection (UTI)
    - Cold and flu
    - Malaria
    - Measles
    - Skin or wound infection
    - Sore throat
    - Body aches
    - Yeast Infection
    - Headaches
    - Syphilis
    - Chlamydia
    - Herpes
    - Human Papillomavirus (HPV)

- COVID-19 (coronavirus)

24. Which of the following are possible side effects after taking an oral antibiotic? (Check all that apply)
    - Nausea/Vomiting
    - Diarrhea
    - Abdominal or stomach pain
    - Headache
    - Rash
    - None of these
    - I'm not sure
    - Other
25. In the past, which of the following places have you gone to get antibiotics? (Check all that apply)
    - Medical Clinic
    - My own supply leftover from previous sickness
    - Neighborhood grocery store
    - Family member or friend
    - Internet pharmacy, or online
    - Community pharmacy
    - Flea Market
    - Swap meet
    - Veterinarian's office or supply store
    - Bodega
    - Health Store
    - I have never taken antibiotics
    - Some other source not listed
26. What do you expect of your clinician during a visit for respiratory illness? (Check all that apply)
    - Symptom relief
    - A prescription for an antibiotic
    - A prescription for a non-antibiotic medication
    - Discussion about appropriate treatment options
    - Testing for flu or COVID-19 (coronavirus)
    - An accurate diagnosis
    - A clear explanation of a diagnosis
    - Reassurance
    - Other

Section 5: Knowledge, Attitudes and Beliefs about Antibiotic Use

In the next section, we will be asking questions about antimicrobial/antibiotic resistance.

Antimicrobial resistance occurs when bacteria change in response to the use of antibiotics used to treat infections making them ineffective.

Please select the answer choice which most closely aligns with your beliefs. (Strongly disagree, Disagree, Neither agree nor disagree, Agree, Strongly agree)

Knowledge, attitudes, and beliefs about antibiotic use

27. Antibiotics should never be saved for the next time you get sick
28. When I have a cold, antibiotics help me to get better more quickly
29. When I have a cold, I should take antibiotics to prevent getting a more serious illness
30. Doctors prescribe antibiotics too often
31. It is okay to borrow antibiotics from friends or family
32. If you requested antibiotics and your physician did not prescribe any, you would be unsatisfied with your physician
33. I trust my healthcare provider's advice as to whether I need antibiotics or not
34. In the big picture of antibiotic resistance, my personal use doesn't matter
35. As soon as you are feeling better, you should stop taking antibiotics
36. Antibiotic resistance is only a problem for those who take antibiotics regularly
37. Skipping or forgetting a dose of antibiotics does not contribute to antibiotic resistance
38. There is no connection between taking antibiotics and the development of resistant bacteria
39. Bacteria that are resistant to antibiotics can be spread from person to person

Section 6: COVID-19 and Antibiotic Use

40. Have you taken antibiotics for flu-like or COVID-19 symptoms since March 2020? (Yes/No)
41. On that occasion, how did you obtain these antibiotics?
    - From a doctor or nurse in a medical setting
    - Online
    - Family member or friend
    - Antibiotics were saved from a previous illness
    - Pharmacy
    - Veterinarian's Office
    - Bodega
    - Health Store
    - Medical Store
    - Flea Market
    - From another country
42. Did you try to obtain antibiotics (ex: azithromycin, also known as a Z-Pak or Zithromax) because of concerns about COVID-19? (Yes/No)
43. Where did you attempt to obtain these antibiotics?
    - From a doctor or nurse at a medical office
    - Internet pharmacy, or online
    - Family member or friend
    - Pharmacy
    - Veterinarian's Office or supply store
    - Bodega
    - Health Store

- Medical Store
- Flea Market
- From traveling to another country
- Some other source not listed

44. Did you try to obtain hydroxychloroquine or chloroquine because of concerns about COVID-19? (Yes/No/I don't know what this is)
45. Where did you attempt to obtain hydroxychloroquine from?
    - From a doctor or nurse at a medical office
    - Internet Pharmacy, or online
    - Family member or friend
    - Pharmacy
    - Veterinarian's Office or supply store
    - Bodega
    - Health Store
    - Medical Store
    - Flea Market
    - Swap meet
    - From traveling to another country
    - Some other source not listed
46. For the latest information on the appropriate treatment for COVID-19, where would you turn? Check all that apply
    - Television News
    - Public health websites like the CDC, WHO, Health Departments, Secretaria de Salud
    - Newspapers
    - Friends and family
    - Facebook
    - Twitter
    - Instagram
    - WhatsApp
    - Your local community pharmacist or other health care provider
    - Other Internet websites (specify)
    - Other (specify)
47. Since COVID-19 has started spreading in my country, I have done the following. (Check all that apply)
    - Stopped working due to being laid off
    - Stopped working (my choice)
    - Continued to work, but from home
    - Continued to work, in person
    - Practiced social distancing in public
    - Practiced hand hygiene more often
    - Worn a mask in public
    - Self-monitored for signs and symptoms of COVID-19
    - Separated myself from others because of symptoms or illness
    - Separated myself from others because of a known or likely exposure
    - Sought out a diagnostic test for COVID-19
48. Have you had a (lab-confirmed) case of COVID-19? (Yes/No)

49. When were you diagnosed with COVID-19?

Please rate the following: (1-7, 1 being not worried at all, 7 being very worried)

50. How worried are you about Coronavirus/COVID-19

Please indicate how likely you think the following situations are: (1-7, 1 being not likely at all, 7 being very likely)

51. How likely do you think it is that you will catch the coronavirus/COVID-19?
52. How likely do you think it is that a friend or family member (in the country you live in) will catch the coronavirus/COVID-19?

Please indicate how strongly you agree or disagree with the following statements: (Strongly disagree, Disagree, Neither agree nor disagree, Agree, Strongly agree)

53. The coronavirus/COVID-19 will NOT affect very many people in the country that I am currently living in
54. I will probably get sick with the coronavirus/COVID-19
55. Getting sick with the coronavirus/COVID-19 can be serious

Please indicate how strongly you agree or disagree with the following statements: (1-7, 1 being not at all, 7 being very much)

56. How much do you trust the country's politicians to deal effectively with the pandemic?
57. To what extent do you feel that the personal actions you are taking to try and limit the spread of coronavirus make a difference?
58. To what extent do you feel the action that your country is taking to limit the spread of coronavirus makes a difference?

Please rate the following:

59. Where do you feel your political views lie on a spectrum of left wing (or liberal) to right wing (or conservative) (very left wing/liberal, left wing/liberal, center left/slightly liberal, middle of the road, center right/slightly conservative, right wing/conservative, very right wing/conservative)
60. How much has your social behavior changed since the COVID-19 pandemic began?
    - Not at all—I still socialize and see others as I always have
    - A little—I try to limit the number of people I'm around from time to time
    - A lot—I try to limit the number of people I'm around most of the time
    - Completely—I have really tried to not be around other people except for my immediate family
61. What are the chances that you will get an FDA-approved COVID-19 vaccine?
    - Almost zero chance
    - Very small chance
    - Small
    - Moderate
    - Large
    - Very large chance

- Almost certain

62. Do you plan on getting the flu shot this year?
    - Yes, I already received the 2020-2021 flu shot
    - Yes, I plan to get the 2020-2021 flu shot
    - No

Section 7: Case Scenarios

63. If a family member were to develop symptoms of mild respiratory infection (congestion, cough) today, what would you do? (Check all that apply)
    - Recommend no change in activities
    - Tell them to stay home and rest
    - Take them to a clinic as soon as possible
    - Take them to a clinic only if symptoms worsened
    - Try to obtain a diagnostic COVID-19 test
    - Seek antibiotics from a doctor or nurse
    - Find antibiotics without having to see a doctor or nurse
    - None of the above
    - Other
64. If you were to develop symptoms of mild respiratory infection (congestion, cough) today, what would you do? (Check all that apply)
    - No change in activities
    - Stay home and rest
    - Go to a clinic as soon as possible
    - Go to a clinic only if symptoms worsened
    - Try to obtain a diagnostic COVID-19 test
    - Seek antibiotics from a doctor or nurse
    - Find antibiotics without having to see a doctor or nurse
    - None of the above
    - Other

Section 8: Healthcare System Questions

The next questions are about your opinion of the health care system in general. When we refer to the health care system, we mean hospitals, health insurance companies, and medical research. For each statement below, please check the extent to which you agree or disagree with the statement. (Strongly agree, Agree, Not sure, Disagree, Strongly disagree)

65. Medical experiments can be done on me without my knowing about it.
66. My medical records are kept private.
67. People die every day because of mistakes by the health care system.
68. When they take my blood, they do tests they don't tell me about.
69. If a mistake were made in my health care, the health care system would try to hide it from me
70. People can get access to my medical records without my approval.

71. The health care system cares more about holding costs down than it does about doing what is needed for my health.
72. I receive high-quality medical care from the health care system. The health care system puts my medical needs above all other considerations when treating my medical problems.
73. Some medicines have things in them that they don’t tell you about

Section 9: Individualism and Collectivism

In the next section we will be asking questions related to your general beliefs. Please check the extent to which you agree or disagree with the statement. (Strongly agree, Agree, Not sure, Disagree, Strongly disagree)

74. I’d rather depend on myself than others.
75. I rely on myself most of the time; I rarely rely on others.
76. I often do “my own thing.”
77. My personal identity, independent of others, is very important to me.
78. It is important that I do my job better than others.
79. Winning is everything.
80. Competition is the law of nature.
81. When another person does better than I do, I get tense and aroused.
82. If a coworker gets a prize, I would feel proud.
83. The well-being of my coworkers is important to me.
84. To me, pleasure is spending time with others.
85. I feel good when I cooperate with others.
86. Parents and children must stay together as much as possible.
87. It is my duty to take care of my family, even when I have to sacrifice what I want.
88. Family members should stick together, no matter what sacrifices are required.
89. It is important to me that I respect the decisions made by my groups.

Section 10: Demographics

90. Gender (Female, Male, Other)
91. Age
    - 18-24
    - 25-34
    - 35-44
    - 45-54
    - 55-64
    - 65+
92. Ethnicity
    - Hispanic/Latinx or of Spanish Origin
    - Not Hispanic/Latinx or of Spanish Origin
93. Choose one or more races that you consider yourself to be:
    - White
    - Asian
    - Black or African American
    - Native Hawaiian or Pacific Islander

- American Indian or Alaska Native
- Other

94. What is the highest level of school you have completed or the highest degree you have received?
    - Less than high school degree
    - High school graduate (high school diploma or equivalent including GED)
    - Bachelor's degree in college (4-year)
    - Post-graduate degree
95. Are you paid in US Dollars or Mexican Pesos?
    - US Dollars
    - Mexican Pesos
96. Please indicate the answer that includes your entire household income in (previous year) before taxes. If you are unsure, please provide your best guess.
    - Less than $20,000
    - $20,000 to $40,000
    - $40,000 to $60,000
    - $60,000 +
97. Please indicate the answer that includes your entire household income in (previous year) before taxes. If you are unsure, please provide your best guess.
    - Less than $10,000 (pesos)
    - $10,000 to $20,000 (pesos)
    - $20,000 to $30,000 (pesos)
    - $30,000 + (pesos)
98. What is the Zip Code (United States) or SEPOMEX (Mexico) of your primary residence?
99. How many people are living or staying in your household, including yourself?
    - 1
    - 2
    - 3
    - 4
    - 5
    - 6
    - More than 6
100. How many children under 16 live or stay in your household?
    - 0
    - 1
    - 2
    - 3
    - 4
    - 5
    - 6
    - More than 6
101. How many seniors older than 65 live or stay in your household?
    - 0

- 1
- 2
- 3
- 4
- 5
- 6
- More than 6

102. Where do you primarily receive healthcare services?
    - United States
    - Mexico
    - I receive services in both countries
103. What best describes your insurance status?
    - Private Insurance (BlueCross BlueShield, Aetna, Humana, Cigna, etc.)
    - Public Insurance (Medicare, Medicaid, AHCCCS, Medi-Cal, Centennial Care, etc.)
    - Uninsured
    - Seguro de Gastos Médicos Privados (Grupo Nacional Provincial, MetLife, etc.)
    - Seguridad Social (IMSS, ISSSTE, etc.)
    - Población Abierta

Section 11: Attention Measurement

104. Please rate how honest you were in your responses to the survey questions today. Please answer honestly and note that your answer will not determine whether or not you receive compensation.
    - Level of honesty: 1 being least honest, 100 being most honest
105. How attentive were you when answering questions today? Please answer honestly and note that your answer will not determine whether or not you receive compensation.
    - Level of attention: 1 being not attentive at all, 100 being most attentive
106. Email address
107. Please enter the email address where you would like to receive your $5 Amazon Gift Card:
108. First and Last Name:
109. How did you hear about this survey?
